# Dilated Odontoma of the Mandible: A Rare Case Report with Discussion about the Differential Diagnosis 

**DOI:** 10.30476/dentjods.2025.106990.2707

**Published:** 2026-06-01

**Authors:** Saede Atarbashi-Moghadam, Ali Lotfi, Nazanin Foroozandehfar, Parsa Eftekhari Moghadam

**Affiliations:** 1 Dept. of Oral and Maxillofacial Pathology, School of Dentistry, Shahid Beheshti University of Medical Sciences, Tehran, Iran.; 2 Student of Research Committee, Shahid Beheshti University of Medical Sciences, Tehran, Iran.

**Keywords:** Dense in dente, Dens invaginatus, Mandible, Impacted tooth

## Abstract

Dilated odontoma is a rare developmental anomaly considered the most severe form of dens invaginatus (dense in dente). It results from a deep enamel-lined invagination into the dental papilla before mineralization, forming a ring-shaped radiopaque mass with a radiolucent center. Anterior mandibular involvement is uncommon. Familiarity with the radiographic features of this lesion is crucial for dental practitioners to make an accurate diagnosis. We report a case of a 12-year-old girl with a painless bony hard swelling in the anterior mandible. Panoramic radiograph revealed a well-defined, doughnut-like radiopaque lesion along with an unerupted permanent canine. The provisional diagnosis was dilated odontoma and ameloblastic fibro-odontoma. The treatment plan consisted of surgical removal of the lesion, followed by histopathologic study to confirm the initial diagnosis. Dilated odontoma may lead to permanent tooth impaction, particularly in atypical locations such as the mandible. It is recommended that the order of tooth eruption in the mixed dentition age group be carefully evaluated.

## Introduction

Dens invaginatus (dens in dente) is a tooth developmental anomaly that shows an invagination of the enamel organ into the dental papilla. This anomaly can affect permanent, deciduous, or supernumerary teeth [ 1 ]. The most commonly affected teeth are the permanent maxillary lateral incisors [ 2 ]. It is divided into three categories based on the depth of enamel-lined invagination. In type 1, the less severe and more frequent form, the invagination is restricted to the crown. In types 2 and 3, the invagination extends beyond the cemento-enamel junction, either remaining inside (type 2) or penetrating through (type 3) the root. In the latter case, this leads to the formation of an additional apical or lateral foramen [ 1 ]. Dilated odontoma represents the most severe manifestation of dens invaginatus [ 3 ]. It arises from a deep infolding of the outer tooth surface into the dental papilla during early tooth development, which may occur in either the crown or the root [ 2 , 4 ]. The lesion shows an approximately spherical mass that lacks the typical structure of a tooth; nevertheless, it displays a tooth-like appearance on radiographs due to its similar radiodensity [ 3 ]. This invagination results in the formation of teeth with abnormal morphology, leading to the formation of a spherical or ovoid calcified structure resembling a doughnut feature [ 1 ]. Accurate analysis of radiographic findings is vital to confirm correct diagnosis, avoiding potential physiological, aesthetic, and functional concerns [ 5 ]. Clinically, these lesions are usually asymptomatic and may remain undetected until they disrupt the normal eruption sequence. In most cases, they are identified either incidentally during routine radiographic evaluations or on discovering the etiology of delayed tooth eruption [ 6 ]. 

This paper presents a rare case of dilated odontoma affecting the left anterior side of the mandible of a 12-year-old girl and describes the clinical, radiographic, and histopathologic characteristics along with a discussion about the differential diagnosis. 

## Case Presentation

**Figure 1:**
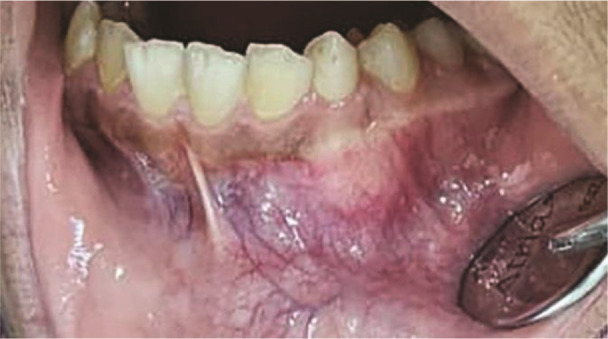
Mild expansion of the left canine and first premolar area with intact mucosa

**Figure 2:**
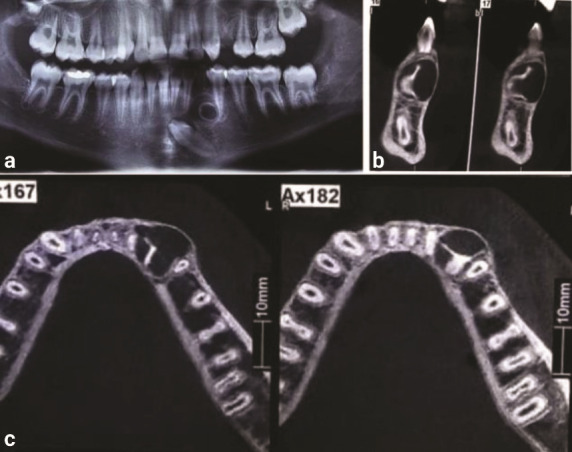
**a:** Panoramic radiography shows a ring-shaped (doughnut-like) radiopacity, associated with a radiolucent area in the center and periphery, **b:** Cone beam computed tomography (CBCT) sagittal view and, c: Axial view

**Figure 3:**
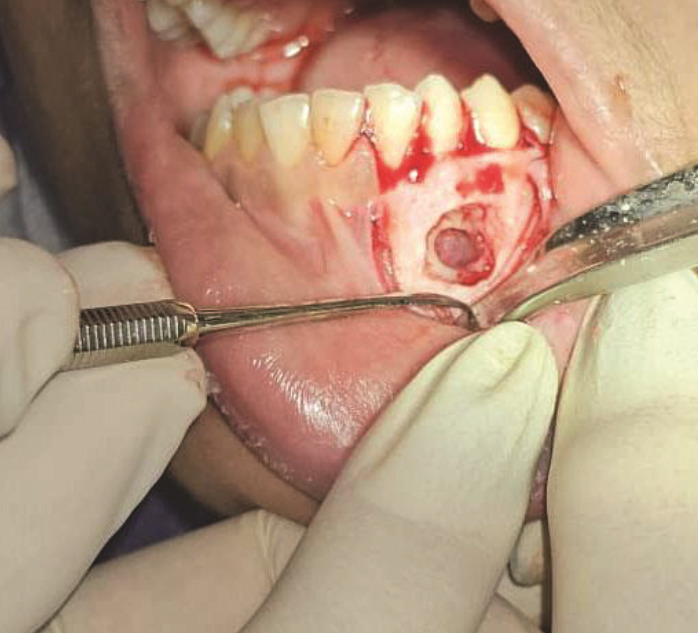
Gross appearance of the lesion during surgery

**Figure 4:**
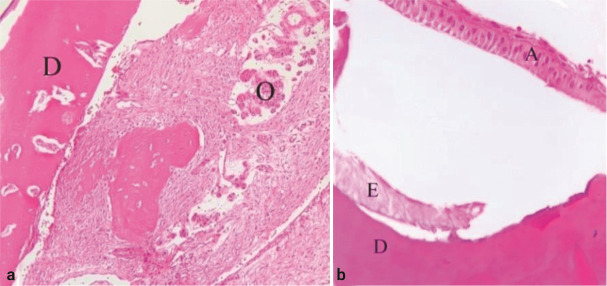
In microscopy, **a:** The outer wall is dentin (D) and the inner side is composed of loose connective tissue with inactive odontogenic islands (O) (H & E, ×100), **b:** Dentin (D), delicate enamel matrix (E) and ameloblasts cells (A) (H & E, ×400)

A 12-year-old girl presented with the chief complaint of mild swelling in the left side of the anterior mandible (Figure 1). Extraoral examination showed no abnormalities, and she reported no significant dental, surgical, or traumatic history. The swelling was non-tender with a bony hard consistency at the apical of the primary left mandibular canine. Except for the absence of the left mandibular permanent canine, the rest of the permanent teeth had erupted, and the number of teeth was normal. The overlying mucosa exhibited a typical appearance, devoid of discernible secondary alterations or fistulous tracts. No lymph nodes in the region were palpable or painful. The panoramic radiograph revealed an oval, ring-shaped (doughnut-like) radiopacity resembling dental structures, associated with a radiolucent area in the center and periphery. The root of the primary canine was partially resorbed, and the impacted permanent mandibular left canine was located apical to the lesion with malposed orientation (Figure 2). The lesion was surgically excised with a differential diagnosis of dilated odontoma, ameloblastic fibro-odontoma (AFO), and odontoma with dentigerous cyst. After removal of the cortex, the lesion showed a bowl-like appearance (Figure 3). Histopathological sections with hematoxylin and eosin (H & E) staining displayed mature dentin, delicate enamel matrix, and pulpal tissue containing inactive odontogenic islands (Figure 4). Based on the clinical, radiographic, and histopathological findings, the dilated odontoma diagnosis was confirmed. The patient is under regular follow-up. 

## Discussion

Dilated odontoma is believed to occur during the early stage of morpho-differentiation. This early disruption is thought to prevent the normal formation of identifiable crown or root anatomy, resulting in a single, calcified, ring-shaped structure [ 4 , 7 ]. Our English literature review of dilated odontoma (just a severe form of dense invaginatus with doughnut-shape appearance) resulted in 20 cases from 13 articles, including our case. The demographic features are summarized and presented in Table 1. Dilated odontoma occurred most often in the second and third decades of life. The mean age was 26.76 (ranging from 7 to 60 years). There was a slight male predilection (M/F=11/9). The most common site was posterior of the mandible (47.05%), followed by posterior maxilla (23.52%), anterior maxilla (17.64%), and anterior mandible (11.76%). About 56.25% of cases were without pain or swelling, and 41.66% of cases were associated with an impacted tooth. Our case presents a rare manifestation of dilated odontoma in the anterior of the mandible, a site that is particularly uncommon for this lesion and contributes to the diagnostic challenge and clinical significance of the case.

**Table 1:** Demographic data of dilated odontoma (severe form of dense invaginatus) reported in the literature

No	Author/year	Age/ Gender	Lesion location	Site	Clinical findings	Subjacent tooth impaction	Other findings
1	Meneses *et al*. [5]/ 2024	-/M	Mandible	NA	Asymptomatic	NA	-
2	Meneses *et al*. [5]/ 2024	-/M	Mandible	NA	Asymptomatic	NA	-
3	Meneses *et al*. [5]/ 2024	-/M	Maxilla	NA	Asymptomatic	NA	-
4	Santos *et al*. [8]/ 2023	32/M	Mandible	Posterior	pain and swelling	No	Proximity with inferior alveolar nerve
5	Galvez *et al*. [10]/ 2021	7/F	Mandible	anterior	delayed eruption	Lateral incisor	-
6	Zara *et al*. [1]/ 2021	11/M	Mandible	Posterior	Asymptomatic	third molar	-
7	Almeida *et al*. [15]/ 2016	47/F	Mandible	Posterior	Pain and discomfort/ slight expansion	No	-
8	Jayachandran *et al*. [3]/ 2016	16/M	Mandible	Posterior	Swelling	No	The lesion was associated with dentigerous cyst and Root resorption
9	Jayachandran *et al*. [3]/ 2016	24/M	Maxilla	Posterior	Occasional pain and swelling	No	Pushing the floor of the right maxillary sinus
10	Syed *et al*. [14] / 2015	23/M	Maxilla	Posterior	Asymptomatic	Third molar	-
11	Sharma *et al*. [4] /2015	18/M	Maxilla	anterior	Malformed Tooth	Central incisor	Erupted DO
12	Sebastian *et al*. [2]/ 2013	30/F	Maxilla	anterior	Swelling and periodic pain	No	Bilateral DO, hypodontia and peg laterals
13	Mahmoodi *et al*. [6]/ 2012	22/F	Maxilla	anterior	Swelling	No	bilateral radicular cysts associated with bilateral, erupted dilated odontomas
14	Matsusue *et al*. [7]/ 2011	14/F	Mandible	Posterior	Unerupted second molar tooth	No	-
15	Cˇukovic´-Bagic *et al*. [16]/ 2010	28/F	Mandible	Posterior	Asymptomatic	No	-
16	Matsumoto *et al*. [13]/ 1996	60/M	Mandible	Posterior	NA	NA	-
17	Matsumoto *et al*. [13]/ 1996	30/M	Mandible	Posterior	NA	NA	-
18	Matsumoto *et al*. [13]/ 1996	49/F	Maxilla	Posterior	NA	NA	-
19	Matsumoto *et al*. [13]/ 1996	32/F	Maxilla	Posterior	NA	NA	-
20	Present case /2025	12/F	Mandible	anterior	Mild swelling	Canine	Root resorption of primary canine

Association with cystic lesions, such as dentigerous cyst, has also been reported [ 3 ]. Suggested etiologic factors have included increased localized external pressure, delayed or accelerated growth of the inner enamel epithelium in specific regions of the tooth bud, infection, trauma, and genetic factors [ 1 , 3 , 8 ]. The mineral composition of the hard tissue may be altered due to this anomaly. However, microscopic differentiation remains relatively intact in cases of dilated odontoma [ 7 ]. The presence of such lesions may interfere with permanent teeth eruption, which therefore results in tooth impaction or malposition, consequently leading to malocclusion and esthetic problems [ 9 , 10 ]. Histologically, the lesion typically exhibits an inverted structure of hard tissues due to the severe invagination of the enamel organ into the developing dental papilla [ 3 ], with an external layer of dentin and enamel forming a ring-like shape, enclosing a central area that often consists of fibrous or pulpal tissue and occasionally contains bone or cementum [ 7 ]. Despite this inversion, the individual dental tissues retain their histological characteristics, aiding in accurate diagnosis [ 3 , 7 ].

From a histopathological perspective, the features supporting a diagnosis of AFO should include a distinctly defined area of ameloblastic fibroma, characterized by narrow drumstick-like cords or islands of ameloblastic epithelium and ectomesenchyme resembling dental papilla. The stromal component should be hypercellular, evenly distributed among the epithelial components, and lack any lobular architecture. Dentin and enamel production should occur randomly within the ameloblastic epithelium areas. In contrast, odontomas typically exhibit compartmentalization, with large areas of hard tissue production located in the center of the lesion, while the ameloblastic epithelium-like changes are observed at the periphery. Small ameloblastic fibroma-like regions with lobular arrangements and hypercellular zones that are only around the epithelium near mature odontomas should not be considered as AFO [ 11 ]. Odontoma is treated by simple excision, and AFO is usually treated by conservative curettage; both have an excellent prognosis and same treatment. If recurrence occurs, a more extensive treatment procedure is indicated for AFO [ 12 ].

Several studies have indicated that the term “dilated odontoma” may be misleading and should be reconsidered [ 1 ]. Matsumoto *et al*. [ 13 ] noted that dilated odontoma is between dense invaginatus and complex odontoma.

Although the lesion is sometimes described as a hamartoma reflecting a developmental defect, it does not represent a true hamartomatous tumor composed of disorganized dental tissues, as seen in classical odontomas [ 14 ]. Instead, the result is a morphologically abnormal tooth structure, often presenting as a spherical or ovoid calcified mass with a radiolucent center. This interpretation aligns with the current WHO classification of odontogenic tumors, which does not recognize dilated odontoma as a distinct pathological entity within the spectrum of odontogenic tumors or odontomas. Therefore, terms such as “dilated dens invaginatus” or “dilated hamartoma of the tooth” may more accurately reflect its developmental origin [ 1 ].

Advanced imaging techniques such as cone beam computed tomography (CBCT) are valuable tools in the diagnosis of dilated odontoma. These allow better visualization of the internal architecture and precise assessment of the lesion’s relationship with adjacent anatomical structures [ 3 , 7 ]. Dilated odontoma may present with variable radiographic appearances, which can complicate the diagnostic process and lead to confusion with other odontogenic lesions. Several lesions, including osteoma, odontoma, osteoblastoma/osteoid osteoma, and ossifying fibroma, have been considered in the radiographic differential diagnosis [ 15 - 16 ]. 

Despite this variability, the histological architecture of the lesion is typically well preserved, and microscopic examination remains essential for establishing a definitive diagnosis [ 3 ]. Furthermore, given the potential of such lesions to disrupt tooth eruption or contribute to esthetic and functional problems, a multidisciplinary approach involving oral radiologists, maxillofacial surgeons, and orthodontists is recommended for comprehensive management [ 4 ].

## Conclusion

Dilated odontoma is a rare odontogenic lesion that can lead to permanent tooth impaction. This case emphasizes the importance of radiographic evaluation in children with altered eruption patterns and presents a rare occurence of dilated odontoma in the anterior mandible, a location with very few documented cases in the literature.
